# The Clinical Society of Maryland

**Published:** 1899-12

**Authors:** 


### SOCIETY REPORTS.

### THE CLINICAL SOCIETY OF MARYLAND.

BALTIMORE, October 20, 1899.

The meeting was called to order by the newly elected President, Dr. James M. Craighill, who made a brief address which embodied a number of suggestions concerning the future work of the society.

The topic for the evening's consideration was "Yellow Fever," and Dr. Francis T. Miles opened the discussion with remarks on the "Clinical Aspects of the Disease."

*Dr. Miles:* It is rather a pretentious thing to put me on the card for a talk on the clinical aspects of this disease when I have not seen a case before this one for a long time. I once saw a great deal of yellow fever, having passed through several epidemics and having had the disease in a mild form myself, but that was a long time ago when I was a medical student. I may say here that I do not remember ever seeing two epidemics occur in successive years, although I am sure the disease was regularly imported into the city of Charleston, S. C., where I resided, every year.

I have seen many post-mortems, but at the time I speak of there was no question of toxic organisms and the disease was considered to be an inflammation of some organ or other. In looking at this case I was forcibly reminded of what I had seen of cases of yellow fever, and the appearances that struck me most, and I can hardly say that I could impart the impression they made upon me to others, were the stupid look about the face, the peculiar expression of the eyes, the reddish yellow color of the skin and the appearance of the black vomit. The mode of vomiting too, that projectile vomiting, appeared very characteristic of the disease. This whole picture impressed me as being that of yellow fever, and I was more certain of it when I heard where the patient had come from.

It might be of some interest for me to say a few words about yellow fever as I saw it many years ago. The fever was characterized almost always by a stormy entrance, the face very much suffused, the eyes glazed and congested, and this condition would last for two or three days, when with a certain amount of suddenness the fever would diminish, the patient become calm, and this condition might be followed almost immediately by a state of collapse, with change of complexion and an appearance that tended to show the peculiar yellow discoloration, and then, almost invariably in bad cases, the disturbance of the stomach began. Indeed, this last symptom was looked for as the most important in relation to the treatment of the disease, for as long as the patient did not vomit the case was considered to be progressing favorably; many cases, however, died without vomiting. The patient might continue in this condition for a time and then a second fever came on; or, as occasionally happened, the fever seemed to absolutely cease, the patient got into a condition that deceived every one into the belief that he was convalescing, and then suddenly there was collapse and death.

At the time I speak of the disease was generally considered as an inflammation of the stomach, and I remember to have seen men supposed to be learned in medicine scrape off the mucous membrane of the stomach and exhibit it as the inflamed stomach, the seat of the disease. At that time I made a number of sections of these stomachs, and I never failed to find the cylindrical epithelium in position, as well as the peptic glands and coils.

With regard to the vomiting, it seemed rather remarkable that the evacuation of the stomach seemed to go on as by a peristaltic action. I have seen a patient in the morning repeatedly gulping and knew that before night he would be vomiting. The peculiar projectile character of this vomiting is very striking. The fluid will be thrown for a distance of one or two yards, and with so little feeling of nausea that the patient would look up and say to the doctor, does that mean anything bad, sir?

In a large number of cases there will be a vomiting of gastric juices, what we called at that time white vomit, hours before the black vomit came on; there was evidently an increased secretion in the stomach. A hemorrhagic condition is usually very marked. In that day, holding to the idea of its being an inflammation, the tendency would have been to treat it by blisters, but we did not dare to do so because any abrasion of the surface produced a hemorrhage that was very hard to control. I have seen hemorrhage from every mucous membrane of the body.

EXHIBITION OF SPECIMENS.

*Dr. Ruhrah:* Before showing the specimens of this case, which died recently at Quarantine Hospital, I want to speak of the general post-mortem occurrences of yellow fever, and since it is such a rare disease here in Baltimore, it may refresh your minds to look at these post-mortem descriptions in some of the older books, which are in many respects better than those of the more recent text-books.

In the cases seen at autopsy there is generally either slight yellow, or more or less orange-colored pigmentation of the skin, sometimes limited to the face and neck, but more generally spread over the trunk and limbs. This pigmentation varies very much, and in those cases that die early the color is lighter, while in those that linger for some time the color is more like mahogany, or even approaches black. In some cases that show no jaundice during life, the yellow color is distinct after death; the same is true of hemorrhages, frequent ecchymosis being found in the tissues after death. The yellow staining may extend to the periosteum, the spinal cord, and even the brain substance itself.

The lungs are usually very much congested, in some cases pneumonia is present, and in some the lung is perfectly solid. Hemorrhagic infarcts are apt to be found near the base of the lungs. In the heart we may find a pericardial effusion, but generally the only change is a degeneration of the muscle. In the late cases we may find the heart so softened and friable that it could be crushed between the fingers, and this is also true of other muscles of the body. The liver is the organ that suffers most. In most cases it is congested, but is about normal in size. There is a general idea that it is friable, but entirely too much stress has been laid upon that point. The organ is in a state of fatty degeneration, so much so, that if it were tried out as much as 50 per cent. of its weight can at times be obtained in pure fat. The characteristic point, however, is the necrosis. In the necrotic area the central portion is yellow, while the peripheral vessels are much enlarged. The spleen is seldom enlarged in cases of mixed infection, but in the simple cases may be slightly enlarged. The stomach and intestines show marked changes, the former being filled with black vomit, the mucous membrane covered with a reddish brown substance, and if this is washed off we find the surface beneath it of a gray slate color. There is generally some engorgement, but in some cases the stomach is paler than normal. There is generally a marked inflammation in some portions of the stomach. The kidneys almost always show marked changes, such as are expected in any infectious disease, the most marked change taking place in the epithelium of the convoluted tubules and consists of swelling and necrosis.

At the autopsy performed on this man we found the typical picture of yellow fever. The man was slightly jaundiced, there were ecchymoses in the skin and conjunctiva, and on making the head section we found the dura mater and periosteum of the cranium stained yellow. The cerebro-spinal fluid was yellow and the brain substance itself was tinged. The brain was very much engorged and there were numerous hemorrhages. The heart and pericardium were apparently normal. The lungs were very much congested, and there were large hemorrhagic infarcts in the posterior portions of both; I have never seen lungs more congested than these. The liver was about the normal size, and there was no evidence of friability, but it was so fatty that it was impossible to determine any necroses, the sections appearing like one mass of fat. The stomach was full of black vomit, and both it and the small intestine were covered with this mucous-like substance, which, when scraped off left the mucous membrane of a grayish color. The spleen was about of normal size and probably a little congested. The kidneys were large and of a type generally spoken of as the large red kidney. (The gross sections of the organs were submitted for examination.)

EXHIBITION OF MICRO-ORGANISMS.

*Drs. Wm. R. Stokes and L. F. Barker:* Cultures were made from the blood and from the organs obtained at the autopsy described above by Dr. Ruhrah and studied independently by Drs. Stokes and Barker. They were not ready to make a complete and final report, but neither had succeeded in isolating the organism of Sanarelli. They explained the methods of obtaining it and gave an exhibition of its growth on culture media.

The President then announced that Surgeon-General Wymann had been prevented from attending the meeting, but had sent in his stead Dr. Sprague, of the Hygienic Laboratory.

*Dr. Sprague:* The subject under discussion is one of interest to us all. I had the good fortune a year ago to see one case of this disease, but my experience is limited to that, and any knowledge of the disease I may have has been obtained through the kindness of others. Dr. Miles in his remarks on the symptomatology of the disease called to my mind very forcibly a feature of the disease that has been dwelt upon by one whose name almost means yellow fever, though perhaps he is not so well known to you as to the members of the Marine Hospital Corps, I mean Dr. Robert Murray, and that was in regard to the vomiting being a reverse peristalsis, as it were. Dr. Murray's idea was exactly in accord with that of Dr. Miles, and he thinks that in the treatment that if he can only keep the bowels moving he will not have the black vomit, and every one knows how successful he has been in his treatment.

In regard to the appearance that Sanarelli's organism produces upon agar, that seal like appearance, it is very characteristic and very pronounced, and once seen, you will remember it. I have never seen it as a cultural characteristic of any other organism. It is difficult to obtain though, and my own opinion is, that it is a trick of Sanarelli's in the technique. It may depend upon some point in the making of the media, in its alkalinity, or in the way in which the organism is spread upon the agar, but I am sure if we once see Sanarelli do it, we shall be able to secure the same picture.

*Dr. Miles:* I want to submit here the excellent report of this case made in the hospital by Dr. Latiny.

### THE ATTEMPTS THAT HAVE BEEN MADE TO DETERMINE THE CAUSE OF YELLOW FEVER.

*Dr. L. F. Barker:* For years the etiology of yellow fever has been the most absorbing problem in American medicine. In the upper part of South America and the Spanish Main an enormous number of victims are given to the disease every year. All efforts toward curing a disease nowadays are preceded by studies of its etiology. I need not stop to mention the early bacteria that were isolated and supposed to be the cause of yellow fever, for instance, those for which claims were made previous to the work done by Dr. Sternberg, but will begin with his work toward the end of the eighties and the beginning of the nineties. Dr. Sternburg worked for several years over the causation of the disease and its pathology, and undoubtedly did us all a great service by describing so accurately his findings. He isolated several organisms from yellow fever cases, but came to the conclusion when his work was done that he had not isolated the cause of the disease. His bacillus was discovered in about half of the cases examined. He brought large amounts of fresh material, some collected within two hours after the death of the individual, to Baltimore and took some of it with him to Europe, where he made a careful study of it, and also turned some of the tissue over to others for study. One thing he did learn was that if he wrapped pieces of the liver in aseptic cloth and put it in the thermostat over night he could in this tissue afterwards demonstrate large numbers of bacilli, and that experiment has been repeatedly followed up since by others. It is very improbable, of course, even if Sanaralli's bacillus is present in the liver, that the bacilli which multiply in a piece of liver treated in that way are the bacilli of Sanarelli. In a large number of yellow fever cases, as Sanarelli himself says, other organisms are present, and as Sanarelli also states that his organism is very easily killed out by other organisms, it would seem that in such pieces of liver the ones that multiply would be those other than the one described by Sanarelli.

I remember that when I came to the Hopkins in 1891 there was much excitement in the laboratory as Drs. Welch and Councilman were both working on this subject and the amount and quality of the material they had made such study well worth while. I well remember the enthusiasm manifested by Dr. Councilman when he found some curious changes in the liver cells. It was before the time when necrosis was thoroughly understood, and in studying these necrotic areas he thought he possibly had the cause of the disease, that it was some amebic organism, and for some days there was some talk of starting an expedition either to Havana or Rio to see if these ameba could not be obtained and isolated in the living body. A further study, however, by Drs. Welch and Councilman, resulted in clearing up most of these curious phenomena. It was shown to be a degeneration in the liver cells, and since then these changes have become familiar to all who work on the pathology of the liver.

About this time the very remarkable studies of Hobbleberg in Rio were published. He found an organism in the stomach of yellow fever cases, but not in the blood or tissues. It was not well distinguished from the bacillus coli communis and probably was that or a member of that group. This was followed in 1887 by the startling discovery of Sanarelli. He isolated his bacillus in 58 per cent. of his cases, and admitted that in 42 per cent. he could not find it. It was always coexistent with other organisms, but he was fortunate in finding it in the second case examined in almost pure culture. He found it in the tissues and in the blood, but said it was absolutely absent from the alimentary tract, and he came to the conclusion that the infection occurred through the lungs. In a subsequent paper he published some remarkable experiments on men. In the first communication he spoke of the production of the poison which was very injurious to animals, killing them with symptoms of hemorrhage, fatty degeneration and the production of a yellow discoloration. In his second article he described some experiments which we, in an Anglo-Saxon country, would certainly consider criminal. He did not use a living culture of the organism, but the toxin, and after injecting his patients described the phenomena that followed. He is said to have produced first nausea, then black vomiting, hemorrhages, coma, and collapse. It is really blood-curdling to read his description. It is interesting to note that his first article appeared in the annals of the Pasteur Institute, but his second article was not printed there. His report excited great interest throughout the world, and many bacteriologists turned their attention to a study of his investigations. Since then he has produced a curative serum to be used in the treatment of yellow fever. As yet the statistics are not sufficient to tell us how beneficial this will be. His first statistics gave a mortality of 22.5 per cent., a mortality less than that of many epidemics, though there have been epidemics with a mortality lower than that. In his first and second articles Sanarelli said nothing about the agglutinative action of the blood serum upon his bacillus, but later, when the Widal test became so generally employed many investigators tried to employ it in this connection, sometimes obtaining the reaction and sometimes failing.

*Dr. Sprague:* They usually succeeded with dilutions of 1 to 10.

*Dr. Barker:* Which of course is of no practical value. Very soon after Sanarelli's publication Dr. Sternberg read it carefully, looked up his own reports and found that his bacillus X, in many of its characteristics at any rate, corresponded to the bacillus of Sanarelli. He also had found his organism in about half of the cases examined, had found it present with other organisms and believed it a secondary invader, and he came to the conclusion in a long polemic in the *American Journal of Medical Sciences* that if bacillus X and the bacillus icteroids were identical and were the cause of yellow fever, then he discovered it, and if they were not the cause of yellow fever, why that was what he said in 1890. He immediately set two of his scientists, namely, Drs. Reed and Carroll, at work upon bacillus X and the bacillus of Sanarelli. They found that bacillus X was not identical with the bacillus of Sanarelli, but they found that in working with the bacillus of Sanarelli that it constantly reminded them of another bacillus, namely, that of hog cholera. They discovered this when inoculating animals with Sanarelli's organism, and finding that it produced curious lesions in the intestines.

They consulted Dr. Theobald Smith, an expert upon this subject, who thought the organism they were working with might be placed in the hog cholera group. They then obtained the hog cholera bacillus, experimented with it, and so far as they could find, it seemed practically identical with the organism of Sanarelli.

Their report was followed by a lively article from Sanarelli in which he suggested that Drs. Reed and Carroll had mixed up their culture and worked with hog cholera instead of his organism. He urged several differences between the two organisms, and said that the growth of the two upon potato was quite different. He accussed Carroll and Reed of being unfamiliar with the literature of the subject and advised them to study it up, but it turns out now that the bacillus to which Sanarelli was attributing these differences was not that of hog cholera, but the bacillus of swine plague, so they may now return him his own advice.

Finally we have just had published this very interesting report from Havana by Drs. Wasden and Gittings. I believe their report states that out of 14 cases of typical yellow fever the bacillus icteroids was isolated in 13. Of course, that is very strong evidence in favor of Sanarelli's bacillus being the cause of yellow fever, for if we can get it from the blood in 13 out of 14 cases, it looks as if it stands in some causative relation.

---

### Rectal Injections of Saline Solutions in Hemorrhage, Shock and Infection.

Lepine closes a thesis on this subject with the following conclusions: There are two classes of phenomena dependent upon such injections. The first are physical or chemical, and correspond to those incident to blood transfusion or the transfusion of artificial serum. The second are purely physiological and are not clearly understood. Intravenous and subcutaneous injections are not absolutely free of danger, and are somewhat difficult of application. Absorption of the saline solutions by the rectal mucous membrane is as easy and rapid as that which takes place when the solutions are injected beneath the skin. The therapeutic effect of the rectal injections is precisely the same as that of subcutaneous injections. The strength of the solution varies according to the indications.—*Therap. Gazette.*

